# Twin to Twin Transfusion Syndrome: A Case Report

**DOI:** 10.31729/jnma.5574

**Published:** 2022-12-31

**Authors:** Pooja Agrawal, Achala Vaidya, Anshu Vaidya, Subash Phuyal, Asmita Pandey

**Affiliations:** 1 Department of Radiology, Norvic International Hospital, Thapathali, Kathmandu, Nepal; 2 Department of Obstetrics and Gynecology, Norvic International Hospital, Thapathali, Kathmandu, Nepal; 3 Department of Radiology, Grande International Hospital, Tokha, Kathmandu, Nepal

**Keywords:** *case reports*, *fetoscopy*, *oligohydramnios*, *polyhydramnios*, *twins*

## Abstract

Twin-twin transfusion syndrome occurs in multiple gestations and involves a chronic flow of blood from one twin to another twin and is a rare entity. We present a case of 32-years-old primigravida with a twin pregnancy who presented with increasing abdominal girth inappropriate with her gestational age at 21 weeks of her pregnancy. Ultrasound findings were suggestive of twin-twin transfusion syndrome. The patient was provided with treatment options but due to polyhydramnios and short cervix, the patient went into spontaneous labour the same day with a poor pregnancy outcome. Twin-twin transfusion syndrome leads to a high rate of perinatal morbidity due to its poorly understood aetiology and difficulty in diagnosing and treatment. Early diagnosis during antenatal ultrasound is important in reducing morbidity and mortality rates.

## INTRODUCTION

Twin-twin transfusion syndrome (TTTS) occurs in multiple gestations involving a chronic flow of blood from one twin to co-twin with a prevalence of approximately 1 to 3 per 10,000 births.^1^ TTTS complicates 10% to 15% of monochorionic (MC) multiple pregnancies.^2^ This syndrome usually occurs in MC twins who have a very high rate of complications including preterm delivery, foetal growth restriction, foetal death, and TTTS. The morbidity rate of TTTS is high, ranging from 40 to 70%.^3^ Its high perinatal mortality rate has been reduced over the last decade by intrauterine treatment options like serial amnioreduction, laser coagulation, and cord occlusion for selective foeticide improving the survival of the remaining foetus.

## CASE REPORT

**Figure 1:**
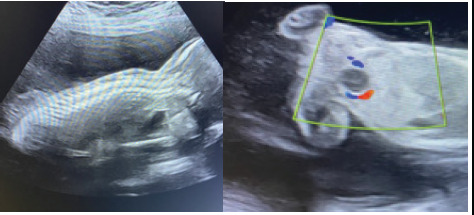
Recipient twin with an average gestational age of 22 weeks 3 days gestation, showing well distended urinary bladder.

A 32-years-old primigravida with twin pregnancy presented at 21 weeks of her pregnancy (Figure 1).

**Figure 2:**
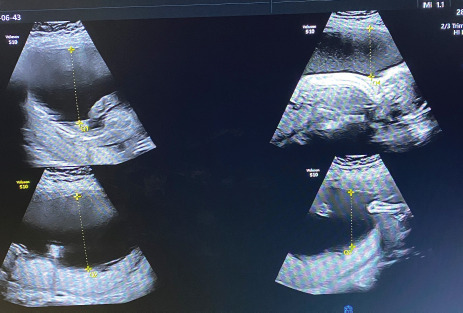
Recipient twin showing with increased liquor volume (single largest vertical pocket >8 cm), AFI of approximately 30.9 cm.

**Figure 3:**
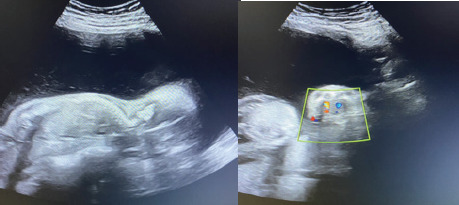
Donor twin foetal biometry corresponding to 19 weeks 5 days of gestation, and appears stuck to a posterior dependent position of the uterus in a prone position with reduced/ almost nil liquor volume, showing an empty urinary bladder.

Her abdominal height was more than the expected 21 weeks pregnancy. The abdominal height was around 34-36 weeks of pregnancy and foetal parts were not palpable. Her vitals were stable. Her blood glucose test and thyroid profile lab tests were unremarkable. The viral markers were non-reactive. She had a previous ultrasound report done at 14 weeks of gestation, which showed monochorionic diamniotic (MCDA) twin foetus with the single posterior placenta. The 14 weeks ultrasound report was unremarkable and there was no significant difference in foetal weight. The patient was referred to us for antenatal ultrasound evaluation. Ultrasound revealed MCDA twin live intrauterine pregnancy with a single posterior placenta. One of the foetuses (foetus A) was free-floating with normal foetal growth and movement. However, the other foetus (foetus B) was stuck to the posterior dependent position of the uterus in a prone position and did not move through the 45 minutes observation period. There was discordant foetal growth and weight with foetus A corresponding to 22 weeks 3 days gestation, weight approximately 486 g, with increased liquor volume, amniotic fluid index (AFI) of approximately 30.9 cm (Figure 2) whereas foetus B corresponding to 19 weeks 5 days of gestation, weight approximately 323 g, with reduced or almost nil liquor volume (Figure 3).

**Figure 4:**
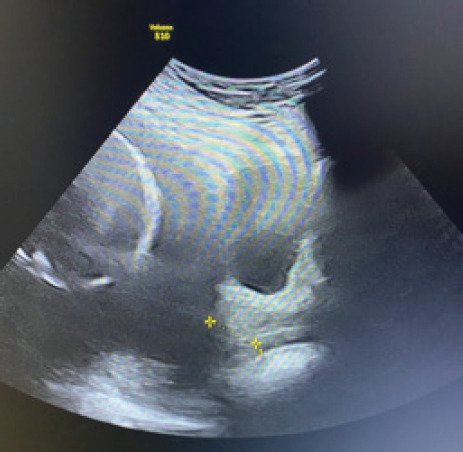
Short cervix of 2 cm.

Foetus A had normal morphological features with well distended urinary bladder. There was no ascites, pleural effusion, nuchal edema however umbilical cord appears edematous. Foetus B was stuck in a posterior dependent position in a prone position with limited morphological evaluation and urinary bladder was not visible throughout the scan. Doppler ultrasound of the umbilical artery was however unremarkable. Based on this, a diagnosis of TTTS (Quintero staging 2) was made with twin A as recipient and B as the donor. The patient also had short cervix with a cervical length of 2 cm (Figure 4).

Based on the ultrasound report, the patient was provided with treatment option. However, the patient went into spontaneous labor the same day later in the evening and delivered foetuses of same-gender with a single placenta. Polyhydramnios with short cervix could have been the predisposing factor. The parents refused further treatment and both the foetus were not resuscitated at the patient's party request.

## DISCUSSION

TTTS is the result of a transplacental shunting of blood from one twin to the other through deep arteriovenous anastomoses. All MC placentas have some degree of vascular anastomoses, but TTTS occurs only in about 15% of MC pregnancies. The donor twin slowly pumps blood to the recipient twin, and failure to compensate for the circulatory imbalance set up by deep unidirectional arteriovenous anastomoses, as the result of a paucity of superficial bidirectional arterio-arterial anastomoses, has been proposed as the pathogenic mechanism.^4^ The degree of TTTS is identified prenatally by ultrasound using Quintero's proposed sonographic staging classification.^5^ The diagnosis of TTTS requires two criteria:(1) the presence of an MCDA pregnancy, and (2) the presence of oligohydramnios (defined as a maximal vertical pocket of <2 cm) in one sac, and of polyhydramnios (a maximal vertical pocket of >8 cm) in the other sac.^7^ Other features that help in diagnosis include a marked difference in foetuses size of the same gender, the difference in the size of the umbilical cord, a single placenta, fluid buildup in the skin of either foetus and findings of congestive heart failure in the recipient twin. The recipient twin is usually appropriate or large for gestational age, shows a distended bladder on ultrasonography, may show signs of cardiac overload upon foetal echocardiogram, and in most severe cases may evolve to hydrops foetalis. The donor twin is often small or intrauterine growth restricted, anemic, and has poor umbilical artery Doppler results. The donor twin is sometimes referred to as the "stuck" twin, because the smaller foetus with minimal fluid appears stuck to the wall of the uterus. Mothers with TTTS may experience a sensation of rapid growth of the uterus, abdominal pain, tightness, contractions, a sudden increase in body weight, swellings in hands and legs in early pregnancy. Complications of TTTS are premature labor either due to ruptured membranes or induction, respiratory, digestive, heart, or brain defects in the recipient twin because of excess fluid and donor twin developing anemia and foetal demise.

TTTS is managed prenatally in an attempt to decrease the rate of morbidity and mortality. The treatment options depend on foetal gestation, placental position, stage of the disease, and geographical location. The two main treatment options are amnioreduction and fetoscopic laser photocoagulation of placental vascular anastomoses. Amnioreduction is the most commonly used and readily available option. The main aim of amnioreduction is to reduce amniotic fluid volume and pressure, thereby reducing the risk of preterm labor or ruptured membranes. However, this technique doesn't address the underlying cause of TTTS. Laser therapy by ablating chorionic plate anastomoses reduces or abolishes intertwin transfusion.^6^ Recent studies comparing amnioreduction and laser coagulation/ ablation have suggested the use of laser ablation as the new standard of treatment. Another treatment option is septostomy, which is creation of microscopic holes in the amnion wall to allow equalization of fluid volume between the twins. The final treatment option of selective reduction, is reserved for TTTS that is severe enough or the recipient twin is near demise due to an advanced stage of cardiomyopathy.^7^

Because of the high-risk nature of the MCDA pregnancy, health-care providers should inform pregnant women with MCDA pregnancy of all the complications and to immediately report in the presence of any symptom described above. We further recommend health-care providers promptly investigate the complaints and physical problems experienced by women during an MCDA pregnancy.
